# Determinants of Response to Cardiac Resynchronization Therapy

**DOI:** 10.19102/icrm.2022.130503

**Published:** 2022-05-15

**Authors:** John D. Allison, Yitschak Biton, Theofanie Mela

**Affiliations:** ^1^The Demoulas Center for Cardiac Arrhythmias, Department of Medicine, Massachusetts General Hospital, Boston, MA, USA; ^2^Cardiac Arrhythmia Service, Hadasssah Medical Center and Hebrew University in Jerusalem Medical School, Jerusalem, Israel

**Keywords:** Cardiac pacing, cardiac resynchronization therapy, heart failure

## Abstract

Cardiac resynchronization therapy (CRT) is a well-established treatment modality for ambulatory patients with heart failure (HF) who have prolonged QRS, left bundle branch block, reduced left ventricular (LV) ejection fraction, and New York Heart Association class II–IV. CRT has been shown to induce reverse LV remodeling and improve HF symptoms and clinical outcomes. About one-third of CRT recipients are considered non-responders. Patient selection, LV lead location, LV lead selection, multipoint pacing, and optimization of the atrioventricular and ventriculo-ventricular intervals were all shown to be associated with a better CRT response rate. Herein, we review the determinants of CRT response.

## Introduction

Cardiac resynchronization therapy (CRT) is a well-established treatment modality for patients with heart failure (HF) from either ischemic or non-ischemic etiology, depressed left ventricular (LV) ejection fraction (LVEF), and evidence of electrical dyssynchrony. CRT has also been shown to be beneficial in patients with mildly reduced LVEF who have an indication for pacing and are expected to have a high burden of right ventricular (RV) pacing.^1–3^ Pivotal studies have shown beneficial effects in terms of morbidity and long-term mortality with CRT. The rate of CRT non-responders varies among studies (25%–33%) depending on the definition of “non-responder.”^4–9^ There is currently no consensus on the definition of what constitutes a CRT responder. The guidelines recommend CRT based on the data that show a reduction in death and HF hospitalizations, but these are not practical metrics to assess on the patient level. Objective echocardiographic findings of reverse remodeling, like LVEF and LV end-systolic volume (LVESV), can be used to gauge the response to resynchronization, but these are not reliable predictors of improved morbidity and mortality.^10^ Improvements in HF symptoms, as assessed by changes in New York Heart Association (NYHA) class, have been correlated with survival, but this is a subjective variable that can be difficult to consistently measure.^10^

In this article, we aim to review the current literature and summarize the clinical, pre-procedural, intra-procedural, and post-procedural determinants of a favorable response to CRT.

## Patient selection

It is imperative to carefully choose the patients most appropriate for CRT placement. Current clinical guidelines recommend a hierarchy of patient characteristics when choosing appropriate patients for CRT that reflect data from clinical trials and analyses.^1,2^ These characteristics are QRS duration, QRS morphology, cardiac rhythm, and expected pacing burden. Additionally, all patients should be on maximally tolerated guideline-directed medical therapy for ≥3 months with no other reversible etiologies of cardiomyopathy (eg, ischemia, atrial fibrillation [AF]). These recommendations are summarized in **Table 1**.

It goes without saying that the ultimate goal of CRT is to improve the NYHA functional class and reduce cardiovascular mortality by reversal of ventricular dyssynchrony. As ventricular dyssynchrony is not regularly quantified, the most readily available surrogate for ventricular dyssynchrony is the surface electrocardiogram (ECG), and randomized controlled trials for CRT have historically used QRS duration (but not morphology) to determine enrollment. As such, the most compelling evidence for CRT is in patients with a QRS duration of ≥150 ms. Much of the evidence for CRT based on QRS morphology (left bundle branch block [LBBB] vs. right bundle branch block [RBBB] vs. intraventricular conduction delay) is gleamed from post-hoc analyses, registry studies, and meta-analyses. Zareba et al. showed in the Multicenter Automatic Defibrillator Implantation Trial—Cardiac Resynchronization Therapy (MADIT-CRT) that, among patients with mild HF, a LVEF of ≤35%, and QRS duration of ≥130 ms, only patients with LBBB derived clinical benefit with CRT with defibrillation (CRT-D).^11^ Similarly, Gold et al. showed in the Resynchronization Reverses Remodeling in Systolic Left Ventricular Dysfunction (REVERSE) trial that longer QRS duration and LBBB were predictors of better reverse remodeling.^12^ Sipahi et al. showed in their meta-analysis that only patients with a QRS duration of ≥150 ms experienced clinical benefit with CRT. However, the investigators did not stratify patients by the QRS morphology.^13^ In a U.S. Food and Drug Administration patient-level meta-analysis, it was shown that women with LBBB had better outcomes than men with a QRS duration of 130–150 ms and similar outcomes with a QRS duration of ≥150 ms. MADIT-CRT sub-analyses showed that patients with non-LBBB morphology had sustained clinical benefit if they had a P–R duration of >230 ms.^14,15^ It should be noted that, while patients with LBBB and a QRS duration of ≥150 ms are the most likely to benefit from CRT on a population-based scale, there is still variability among individual responders. Some patients with LBBB and a QRS duration of ≥150 ms will not respond, and some patients with RBBB will respond. These findings likely represent the heterogeneity of ventricular dyssynchrony and the limitations of using the surface ECG to assess mechanical dyssynchrony. Relative electrical conduction delay, and thus ventricular dyssynchrony, can occur on multiple levels within the heart, including the atria, atrioventricular (AV) junction, and ventricles. The etiology of the conduction delay can vary as well, including myocardial scar or ventricular dimensions. Such factors can be barriers to CRT response. For example, a patient with LBBB and a QRS duration of ≥150 ms may have myocardial scar at the site of the LV lead, and no amount of pacing from this area can overcome the conduction delay. Importantly, CRT use in patients with a QRS duration of ≤120 ms was associated with adverse outcomes, and these devices should be avoided in patients with narrow QRS.^16^

Baseline rhythm can also affect CRT response rates. Patients with AF were often excluded from the original CRT randomized trials out of concern that AV dyssynchrony would confound the results. From a practical perspective, achieving an optimal biventricular (BiV) pacing percentage during AF can sometimes be difficult due to rapid heart rates. But while up to one-third of patients with HF who would otherwise qualify for CRT have AF, the evidence for these patients is relatively sparse and inconsistent. A randomized trial of standard implantable cardioverter-defibrillator (ICD) versus CRT-D therapy in patients with AF and HF showed no statistically significant reduction in death or HF hospitalization,^17^ and this finding was also confirmed with a meta-analysis.^18^ The data comparing CRT results in patients with sinus rhythm (SR) versus AF are also lacking and inconclusive, with no large prospective randomized trials able to provide guidance. In a subgroup analysis of a randomized trial, patients with AF benefited from CRT, just to a lesser extent than their SR counterparts.^19^ In a subsequent meta-analysis of only prospective studies examining CRT in patients with SR versus AF, there was no difference in mortality or NYHA class, but patients with AF demonstrated a slightly greater improvement in LVEF than those with SR.^20^

Several other factors have been shown to be associated with an improved benefit with CRT, including female sex and a non-ischemic etiology of cardiomyopathy.^21–24^ It should be noted that female sex was correlated with non-ischemic disease, and this association should be taken into account when interpreting those studies. Baseline P–R duration was shown to be inversely related to clinical outcomes. Interestingly, a study showed that, in patients with a non-LBBB configuration, a P–R duration of ≥230 ms identified responders.^14^ This observation should be confirmed in prospective studies.

Some risk scores have been proposed to help predict CRT response that use readily available patient characteristics (like QRS duration, QRS morphology, sex, cardiomyopathy etiology, and LV size, to name a few).^25,26^ At best, these unproven scores provide helpful prognostic information for patients, but none have been externally validated or tested in a prospective study.

With the availability of large databases containing vast amounts of information, machine learning (and perhaps, eventually, artificial intelligence) algorithms are being developed to help predict CRT response. Feeny et al. identified 9 readily available patient characteristics (QRS morphology, QRS duration, NYHA class, LVEF, LV end-diastolic diameter, sex, ischemic cardiomyopathy, AF, and epicardial LV lead) and applied a supervised machine learning program to a training cohort.^27^ Once training was complete, the algorithm was applied to a test cohort, which not only successfully predicted CRT response better than the previously identified clinical predictors as well as the guidelines but also long-term survival. As of now, machine learning has only been used to prognosticate CRT response in a retrospective manner; no prospective investigations have been published. Additionally, while the possibilities for machine learning are exciting, this tool has not been tested in a randomized fashion against guideline-based practice to improve CRT response rates.

## Intra-procedural considerations

**Figure 1:**
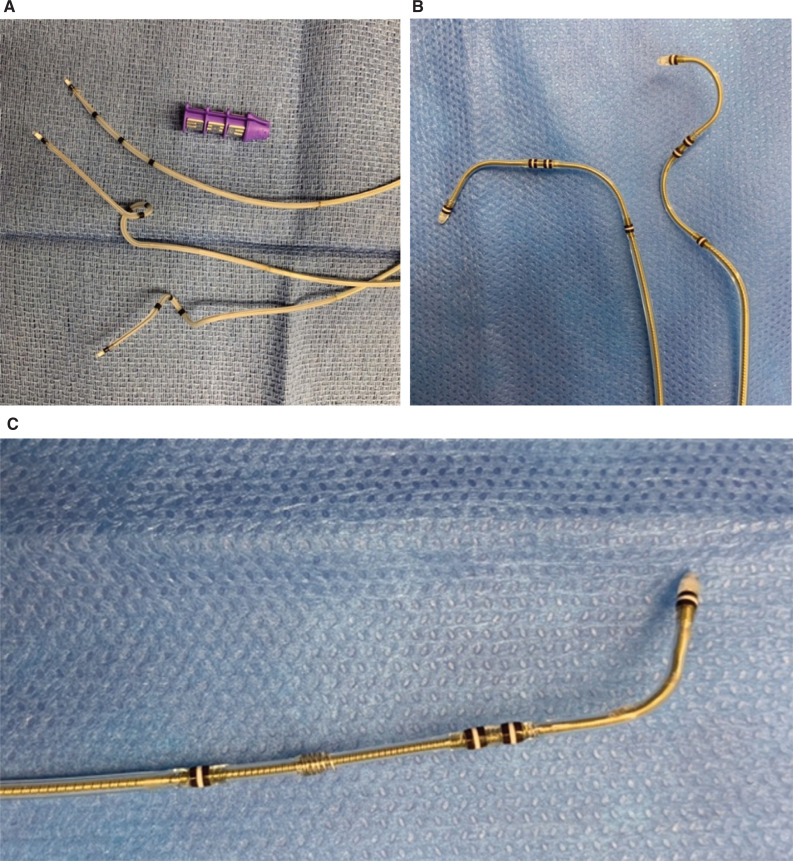
**A:** Boston Scientific shaped quadripolar leads. **B**: Medtronic shaped quadripolar leads. **C:** Medtronic active fixation quadripolar lead.

Anterolateral, midlateral, and posterolateral LV lead locations have been shown to be associated with improved clinical outcomes.^28–30^ Sub-analyses of randomized trials demonstrated that the apical lead position was associated with an increased risk for HF/mortality compared to the basal and mid-ventricular positions. However, the design of early unipolar and bipolar LV leads frequently called for wedging the leads in an apical location to prevent dislodgment, limiting the ability to pace non-apical sites. For this reason, pre-shaped curves and quadripolar lead designs have almost completely replaced the older generations of leads **(Figure 1)**. These lead tips can be advanced to the terminal branch, and proximal electrodes may be used for pacing. Several studies have shown lower dislodgment rates, better thresholds, and lower rates of phrenic nerve stimulation with quadripolar leads compared to unipolar and bipolar leads.^31–35^ The more recent release of an active fixation quadripolar LV lead allows for even greater flexibility in the pacing site and reduces the risk for dislodgment.^36^ However, currently, no study has demonstrated improved objective endpoint outcomes, such as mortality, HF, or LVEF improvement with quadripolar leads compared to older-generation leads.

The use of quadripolar leads to perform multipoint pacing (MPP) has been investigated with reassuring results.^37–40^ With the MPP system, dual-site LV pacing can be performed with 2 different pacing vectors from the quadripolar lead. The More Response on Cardiac Resynchronization Therapy with Multipoint Pacing (MORE-CRT MPP) study was a prospective randomized trial that compared MPP to conventional pacing in CRT non-responders; ultimately, MPP did not result in an improved echocardiographic response (improved LVEF and LVESV), but a subgroup of the MPP arm (named MPP-AS) that was programmed with a wide LV electrode anatomic separation (≥30 mm) and shortest timing delays (5 ms) did experience higher rates of echocardiographic response.^41^ Separately, the MORE-CRT MPP phase II trial sought to assess the 6-month impact of MPP programmed to mandate MPP-AS settings in subjects who do not respond to 6 months of BiV pacing (note, the trial was later terminated in November 2021 by the steering committee due to low probability that the results would meet the primary outcome).^42^

Local intracardiac electrograms have been utilized to identify the site of the most delayed LV electrical activation, which can be preferably selected when possible for LV pacing. QLV measurement, defined as the interval from the onset of the QRS surface ECG to the first positive or negative peak of the LV electrogram, was shown to be highly correlated with clinical outcome, including an improvement in LV dP/dt max and LV reverse remodeling, when stimulating at sites with a QLV delay of >95 ms.^43,44^

Choosing the LV pacing site that results in the surface ECG with the narrowest QRS has recently become a more popular method to identify the preferred LV lead pacing site. Sweeney et al. described that increasing R amplitude in V1–V2, indicating ventricular fusion, was associated with an increased probability of reverse remodeling with BiV pacing.^45^ This finding of increased R-wave prominence in V1/V2, as well as QRS normalization in V1 and V2, and QRS shortening by ≥25 ms were identified as predictors of reverse remodeling in a later study.^46^ These results can be achieved by modifying the lead position during implant or the LV offsets during follow-up.

Body surface mapping using an ECG belt to characterize electrical heterogeneity (EH) for different LV pacing sites during CRT is a newer methodology that has been found to improve the acute hemodynamic response to CRT.^47^ Two EH metrics, the standard deviation of activation times and the mean left thorax activation times, are computed from isochronal maps based on 53-electrode surface mapping during BiV pacing from different sites in coronary veins. The site with the greatest reduction in these 2 parameters was shown to be associated with the greatest hemodynamic response in acute studies. To examine the long-term effect of this type of optimization, the ongoing ECG Belt for CRT Response Study is a prospective, interventional, randomized pre-market study, which uses the ECG belt at implant to help choose the most optimal LV lead pacing site and again at follow-up for vector/timing parameters.^48^

Echocardiographic studies have shown that targeting the areas of latest activated LV segment (concordance) was associated with better LV reverse remodeling. The Targeted Left Ventricular Lead Placement to Guide Cardiac Resynchronization Therapy (TARGET) trial was a randomized study that examined the impact of using baseline echocardiographic speckle-tracking 2-dimensional radial strain imaging versus the conventional approach to guide LV lead positioning. It showed that patients who had guided LV lead placement over a scar-free area experienced fewer HF hospitalizations and better remodeling.^49^ The Speckle-tracking Assisted Resynchronization Therapy for Electrode Region (STARTER) trial randomized patients to guided LV lead placement determining the site of latest time to peak radial strain by speckle-tracking echocardiography versus a conventional fluoroscopy approach. The study showed that the transthoracic echocardiography (TTE)-guided group had a significant reduction in HF and mortality outcomes and better lead placement concordance with the site of latest mechanical activation.^50^

Retrospective magnetic resonance imaging studies have demonstrated an association between LV lead location over transmural scarred areas and CRT non-responders.^51^

The locations of the RV and right atrial (RA) leads have not been shown to correlate with clinical outcomes. Sporadic studies suggested some improvement with septal RV positioning, but these results were not reproducible.^29,52^

## Postprocedural considerations

Most of the responders will experience LVEF improvement and LVESV reduction (reverse LV remodeling) in about 3 months, although it could take up to 2 years. Clinical improvement should also be evaluated with quality-of-life questionnaires and the 6-min walk test.

Close follow-up after CRT implant is important for many reasons. CRT recipients are typically frail patients with HF and other comorbidities. They have different etiologies of cardiomyopathy, including ischemic, non-ischemic, and other concomitant pathologies (RV failure, valvular disease, pulmonary hypertension, etc.). As such, we advocate that this group warrants multidisciplinary care of experts, including electrophysiologists and HF and cardiac imaging specialists. This approach allows for better optimization of the CRT therapy, enables patient education and early intervention for more advanced therapies, and has been shown to be associated with better outcomes.^53^

Prompt response to the information received by the device diagnostics is of great significance. The percentage of BiV pacing is highly correlated with clinical outcome. Every effort should be made to ensure 100% BiV pacing. The best clinical outcomes were shown to be correlated with >98% pacing.^54^ Unfortunately, about 40% of the CRT recipients do not achieve this target.^55^ Supraventricular tachycardia, AF, and ventricular ectopy are known to interfere with BiV pacing. In instances of prolonged AV conduction, shortening of the AV interval should improve the rate of BiV pacing. In instances of enhanced AV nodal conduction, nodal blocking agents will usually suffice. Some algorithms (ie, ventricular sensed response) may provide better synchronization by identifying intrinsic conduction and promoting pacing soon after detection. Two retrospective studies suggested a clinical benefit with AV node ablation in patients with resistant AF, but this strategy should be evaluated in prospective investigations.^56,57^ The Catheter Ablation vs. Standard Conventional Treatment in Patients with LV Dysfunction and AF (CASTLE-AF) trial showed that catheter ablation for AF in patients with HF (27% of patients had CRT devices) significantly reduced the rate of death from any cause and HF hospitalization compared to medical therapy.^58^ If frequent premature ventricular contractions (PVCs) interfere with optimal BiV pacing and cannot be suppressed with pharmacologic treatment, catheter ablation should be considered.^59,60^ In non-responder patients with BiV pacing > 95%, assessment of pseudo-fusion with intrinsic conduction or PVCs is recommended with 12-lead ECG or Holter monitoring, as the rate of BiV pacing may be overestimated.^61^

Optimization of the device to improve the rate of response to CRT has been long disputed, and, even today, there is a lack of consensus as there are insufficient data to support systematic optimization of all patients.^56^

CRT optimization using echocardiographic parameters such as mitral inflow (shortest AV delay without truncation of the A-wave), LV outflow tract velocity time integral, largest stroke volume, tissue Doppler imaging, LV M-mode and septal–lateral wall motion delay, and strain measurements has been extensively studied, but the results have been disappointing.^62^ Early attempts to program an optimal AV and ventriculo-ventricular (VV) delay based on these echocardiographic parameters resulted in a positive acute hemodynamic response.^63^ However, the long-term outcomes were similar with fixed AV delays to patient-specific algorithms and echocardiography-guided approach in both the SmartDelay Determined AV Optimization: A Comparison to Other AV Delay Methods Used in Cardiac Resynchronization Therapy (SMART-AV) study and the Frequent Optimization Study Using the QuickOpt Method (FREEDOM) study.^64,65^ Speckle tracking is an emerging tool in echocardiography that helps define cardiac strain patterns and could potentially find a role in CRT optimization. We have already discussed the results of the STARTER trial that showed better outcomes when strain was assessed intra-procedurally at the time of LV lead placement. Another randomized trial of CRT non-responders, though small with just 30 patients, showed that programming AV and VV delays to optimize strain patterns by speckle tracking resulted in improved LVEF and NYHA class compared to conventional optimization strategies.^66^ There are currently no large, randomized trials testing this method.

Automated device algorithms have been developed simultaneously in an effort to achieve AV and VV optimization in a more continuous and dynamic fashion. These include QuickOpt and SyncAV (Abbott, Chicago, IL, USA); SmartDelay (Boston Scientific, Marlborough, MA, USA); and SonR, MicroPort, and AdaptivCRT (Medtronic, Minneapolis, MN, USA). The Adaptive CRT trial studied the algorithm that enables repeated adjustments of AV and VV intervals based on intrinsic conduction in patients with intact intrinsic conduction (PR ≤ 200 ms).^67^ The investigators demonstrated the non-inferiority of the algorithm compared to TTE optimization at 6 months of follow-up. The ongoing prospective, randomized AdaptResponse trial is examining whether patients with the AdaptivCRT algorithm turned on will have a superior outcome compared to those with standard CRT programming among patients with intact AV conduction and LBBB.^68^ No automated dynamic AV or VV delay algorithms have been shown to be superior to fixed delay programming.

The SonR hemodynamic sensor attempts to optimize the AV and VV intervals based on global ventricular contractility. The Clinical Impact of the SonR Hemodynamic Method (CLEAR) study was a single-blinded study that used an RV lead sensor and showed an increased rate of responders in the sensor arm compared to the standard-of-care arm.^69,70^ The Clinical Trial of the SonRtip Lead and Automatic AV-VV Optimization Algorithm in the PARADYM RF SonR CRT-D (RESPOND-CRT) study included both ischemic and non-ischemic patients with NYHA class III; it showed that sensor-driven optimization of AV/VV intervals was non-inferior to the TTE-guided strategy.^70^ Interestingly, the sensor arm had a lower rate of HF admissions despite no difference in reverse remodeling.

The third method used to optimize CRT is the electrocardiographic QRS-based approach, and it includes the analysis of the 12-lead ECG and fusion-optimized intervals (FOIs). These techniques have become increasingly popular, as they are faster and simpler. A number of older studies have suggested that certain patterns of paced QRS and narrowing of the QRS with CRT can lead to a response to CRT.^46,71,72^ When patients were prospectively randomized to VV optimization with echocardiographic versus QRS width criteria, at 6-month follow-up, the ECG-optimized group had lower mortality and heart transplantation rates and an LVESV reduction of >10% (50% vs. 67.9%, *P* = .0023) compared to the echo-optimized group.^73^

The FOI method uses fusion with intrinsic conduction to achieve the shortest possible QRS and was shown to acutely improve the dP/dT in patients with CRT who have intrinsic conduction and an LBBB pattern.^74,75^ In a 12-month follow-up study, patients randomized to fusion optimization showed a higher rate of reverse remodeling than echo-optimized patients (LVESV was reduced by >15% in 74% vs. 53% of patients, respectively; *P* = .026).^76^ The FOI method identifies first the sensed AV interval that provides the narrowest QRS. It is important to note that the onset of the QRS is the fast deflection and not the pacing spike. The shortest paced AV interval that provides the narrowest QRS is identified next. Then, the VV interval is adjusted during atrial sensing, comparing QRS duration during simultaneous RV/LV pacing, LV pre-excitation by 30 ms, and RV pre-excitation by 30 ms. The VV value that provides the narrowest QRS is considered the fusion-optimized VV interval.^62^ Limitations of the method include patients with AF, complete AV block, prolonged AV interval, and non-LBBB morphology where further research is required. Further research should also include direct comparisons between the echocardiographic, electrocardiographic, and automated device algorithm methodologies to further elucidate what the preferred methodology with the highest patient benefit may be and under what criteria. The role of ECG body-surface mapping also needs to be further defined, as clinical research is in early stages.

Remote monitoring provides a unique way of communication between HF patients and their providers and has become an even more valuable tool during the time of the coronavirus disease 2019 pandemic and the requirement or preference for virtual visits. Early initiatives of remote monitoring focused on patient education, communication with nursing staff by telephone or telemonitoring of patients’ vital signs and weight. Prospective studies failed to show a reduction in HF hospitalizations and mortality of those methodologies compared to routine care.^77,78^ The evolution of remote monitoring included the automated reporting of markers of sympathetic activation and parasympathetic withdrawal—namely, the heart rate variability—among parameters, like arrhythmia occurrence, heart rate, percentage of BiV pacing, patient activity, and intrathoracic impedance. These parameters are used to identify clinical deterioration early and allow preventive treatment. Some studies showed a decrease in hospitalizations, reduced costs, and improvement in patient symptoms.^79–82^

## When cardiac resynchronization therapy fails

One of the most frustrating complications of CRT is the failure to place a coronary sinus (CS) lead. This result is not uncommon and can occur for various reasons—failure to cannulate the CS, absence of CS branches that can accommodate a lead, high pacing thresholds due to scar, diaphragm stimulation from multiple vectors, or unstable lead position, to name a few. Fortunately, several alternatives to CS lead placement exist, including an LV epicardial lead, LV endocardial lead, and left bundle area pacing (LBAP). While there are pros and cons to each approach, none have been effectively compared to conventional CRT in a head-to-head fashion.

Surgically implanted epicardial LV leads have historically been the back-up option when a CS lead is not successful. In a non-randomized prospective cohort of patients who qualified for CRT, in which an epicardial LV lead was surgically implanted when a transvenous CS lead could not be placed, both groups experienced effective narrowing of the QRS from 182 ± 22 ms to 143 ± 16 ms (*P* < .001), with no difference between the 2 techniques.^83^ In a separate retrospective analysis, there was no difference in 90-day mortality between surgical and transvenous LV leads, and, while there was a significant reduction in NYHA class and LV end-diastolic diameter (LVEDD) in the surgical group at 9 months, there was no change in EF.^84^ The most obvious disadvantage of a surgical epicardial LV lead is that it requires a thoracotomy (mini-thoracotomies are available at some centers), but it should also be mentioned that there are no long-term data for HF or mortality available compared to conventional transvenous CS leads. Endocardial LV lead placement has also been attempted. The initial approach was transseptal, and though a statistically significant improvement in LVEF was achieved, the results were marred by thromboembolic events.^85^ The WiSE-CRT system (EBR Systems, Sunnyvale, CA, USA) involves a wireless LV lead implanted via a retrograde or transseptal approach that is powered by a separate subcutaneous generator placed on the patient’s left side. In the phase I trial, improvements in LVEF, LVESV, and LVEDV were noted, but more data are needed regarding long-term safety and efficacy.^86^

Perhaps the most anticipated alternative to CS pacing is LBAP. The technique aims to achieve the same goal as His-bundle pacing (HBP), which is selective pacing of the native conduction system, but promises to overcome the primary shortcomings of HBP that include lead instability and trouble with maintaining low pacing thresholds. In a prospective randomized study of HBP versus conventional CRT, HBP resulted in more improved LVEF and LVESV but was limited by high pacing thresholds at 6 months.^87^ In a non-randomized study comparing LBAP, HBP, and CS pacing, LBAP and HBP were comparable in regard to LVEF improvement and NYHA class improvement, and both were better than CS pacing.^88^ It is only a matter of time before we have results from a prospective randomized trial with long-term efficacy of LBAP versus CS pacing.

## Conclusions

In summary, CRT is a fundamental treatment modality for a selected group of patients with HF. Guideline-based patient selection, intra-procedural measures (LV lead location, optimal AV and VV intervals, utilizing quadripolar leads and advanced imaging modalities in selected patients) and postprocedural measures (maximizing the BiV pacing rate and optimizing the pacing parameters) can decrease the rate of non-responders to a minimum **(Table 2)**.

## Figures and Tables

**Table 1:** Summary of Guideline Recommendations for Cardiac Resynchronization Therapy Based on Baseline Patient Characteristics

		NYHA Class	ACC/AHA/HRS (2013)^1^	ESC/EHRA (2021)^2^
LBBB	QRS ≥ 150 ms	Class III/IV	I	Ia
		Class II	I	Ia
	QRS 130–149 ms	Class III/IV	IIa	IIa
		Class II	IIa	IIa
	QRS 120–129 ms	Class III/IV	IIa	III
		Class II	IIa	III
Non-LBBB	QRS ≥ 150 ms	Class III/IV	IIa	IIa
		Class II	IIb	IIa
	QRS 130–149 ms	Class III/IV	IIb	IIb
		Class II	III	IIb
	QRS 120–129 ms	Class III/IV	IIb	III
		Class II	III	III
AF and HF		Class III/IV	IIa	IIa
Expected high % RV pacing + HF + low EF			IIa (RV pacing ≥ 40%)	Ib–IIa

*Abbreviations:* ACC, American College of Cardiology; AHA, American Heart Association; AF, atrial fibrillation; EF, ejection fraction; EHRA, European Heart Rhythm Association; ESC, European Society of Cardiology; HF, heart failure; HRS, Heart Rhythm Society; LBBB, left bundle branch block; NYHA, New York Heart Association; RV, right ventricle.

**Table 2:** Determinants of Response to Cardiac Resynchronization Therapy with Evidence

	Intervention	Outcome	Evidence
Pre-procedure	QRS ≥ 150 ms	Reduced mortality and HF	RCTs,^4–9^ meta-analysis^13^
	LBBB	Reduced mortality, improved LVEF	Subgroup analyses of RCTs, meta-analyses^11,12^
Intra-procedure	Non-apical lead position	Reduced mortality and HF	Subgroup analyses of RCTs^28–30^
	Multipoint pacing (MPP-AS)	Improved LVEF	Subgroup analysis of RCT^41^
	Maximize QLV	Improved echocardiographic LV remodeling, improved QOL	Sub-study of RCT,^43^ non-randomized prospective cohort^44^
	Maximize QRS narrowing and QRS fusion	Improved LVESV	Subgroup analyses of RCT,^45^ non-randomized prospective cohort^46^
	Optimize LV strain	Reduced mortality and HF. Improved NYHA and LVEF	RCTs^49,50^
Post-procedure	Maximize BiV pacing percentage	Reduced mortality and HF	Post-hoc subgroup analysis of RCT,^89^ non-randomized prospective cohort^90^
	Program delays to optimize LV strain pattern	Improved HF and LVEF	Small RCT^66^
	Fusion-optimized interval	Improved LVESV	RCT^73^

*Abbreviations:* BiV, biventricular; HF, heart failure; LBBB, left bundle branch block; LV, left ventricle; LVEF, left ventricular ejection fraction; LVESV, left ventricular end-systolic volume; NYHA, New York Heart Association; MPP-AS, multipoint pacing with wide anatomic separation; RCT, randomized controlled trial.
